# Trisomy 21 causes changes in the circulating proteome indicative of chronic autoinflammation

**DOI:** 10.1038/s41598-017-13858-3

**Published:** 2017-11-01

**Authors:** Kelly D. Sullivan, Donald Evans, Ahwan Pandey, Thomas H. Hraha, Keith P. Smith, Neil Markham, Angela L. Rachubinski, Kristine Wolter-Warmerdam, Francis Hickey, Joaquin M. Espinosa, Thomas Blumenthal

**Affiliations:** 1 Linda Crnic Institute for Down Syndrome, University of Colorado School of Medicine, Aurora, Colorado, 80045 USA; 2 Department of Pharmacology, University of Colorado School of Medicine, Aurora, Colorado, 80045 USA; 3 SomaLogic, Inc., Boulder, CO 80301 USA; 4 JFK Partners/Developmental Pediatrics, Department of Pediatrics, University of Colorado School of Medicine, Aurora, Colorado, 80045 USA; 5 Anna and John J. Sie Center for Down Syndrome, Children’s Hospital Colorado, Aurora, Colorado, 80045 USA; 6 Department of Molecular, Cellular and Developmental Biology, University of Colorado Boulder, Boulder, Colorado, 80203 USA; 7 Department of Biochemistry and Molecular Genetics, University of Colorado School of Medicine, Aurora, Colorado, 80045 USA

## Abstract

Trisomy 21 (T21) causes Down syndrome (DS), but the mechanisms by which T21 produces the different disease spectrum observed in people with DS are unknown. We recently identified an activated interferon response associated with T21 in human cells of different origins, consistent with overexpression of the four interferon receptors encoded on chromosome 21, and proposed that DS could be understood partially as an interferonopathy. However, the impact of T21 on systemic signaling cascades in living individuals with DS is undefined. To address this knowledge gap, we employed proteomics approaches to analyze blood samples from 263 individuals, 165 of them with DS, leading to the identification of dozens of proteins that are consistently deregulated by T21. Most prominent among these proteins are numerous factors involved in immune control, the complement cascade, and growth factor signaling. Importantly, people with DS display higher levels of many pro-inflammatory cytokines (e.g. IL-6, MCP-1, IL-22, TNF-α) and pronounced complement consumption, resembling changes seen in type I interferonopathies and other autoinflammatory conditions. Therefore, these results are consistent with the hypothesis that increased interferon signaling caused by T21 leads to chronic immune dysregulation, and justify investigations to define the therapeutic value of immune-modulatory strategies in DS.

## Introduction

Trisomy 21 (T21), the molecular cause of Down syndrome (DS), is the most common chromosomal abnormality in humans, occurring in 1 in ~700 live births^1,2^. Individuals with DS display an altered disease spectrum, whereby they are protected from certain diseases, but more prone to others^1,3–6^. For example, people with T21 are highly predisposed to develop autoimmune conditions such as Hashimoto’s disease/hypothyroidism^7^, celiac disease^7,8^, type I diabetes^7,9^, alopecia areata^10,11^, vitiligo^10,11^, and rheumatoid arthritis^12,13^, as well as diverse seizures of unknown etiology that could be linked to chronic neuroinflammation^14,15^. While the genetic underpinning of DS has been known for more than half a century^16^, it is still unclear how the extra copy of chromosome 21 (chr21) causes the various phenotypes observed in individuals with DS. Recently, we reported the results of transcriptome analyses of four different cell types obtained from people with and without T21, which demonstrated that T21 causes widespread alterations in gene expression across the genome, including, most prominently, consistent activation of the interferon transcriptional response^17^. However, the impact of T21 on systemic signaling cascades in living individuals with DS has not been elucidated. To advance this research area, we applied the SOMAscan^®^ proteomics platform to assess hundreds of proteins in small volumes of plasma or serum samples obtained from three independent cohorts of individuals with and without DS. A fourth cohort was analyzed with a different platform to measure key proteins of interest. Collectively, these efforts identified dozens of proteins consistently deregulated in the circulating proteome of people with T21. A key result is that individuals with DS display obvious signs of autoinflammation across their lifespan, many of which strongly resemble those observed in type I interferonopathies and other chronic autoinflammatory conditions, reinforcing the notion that DS could be understood in good measure as an immune disorder.

## Results and Discussion

### Trisomy 21 causes consistent global changes in the circulating proteome

**Figure 1:**
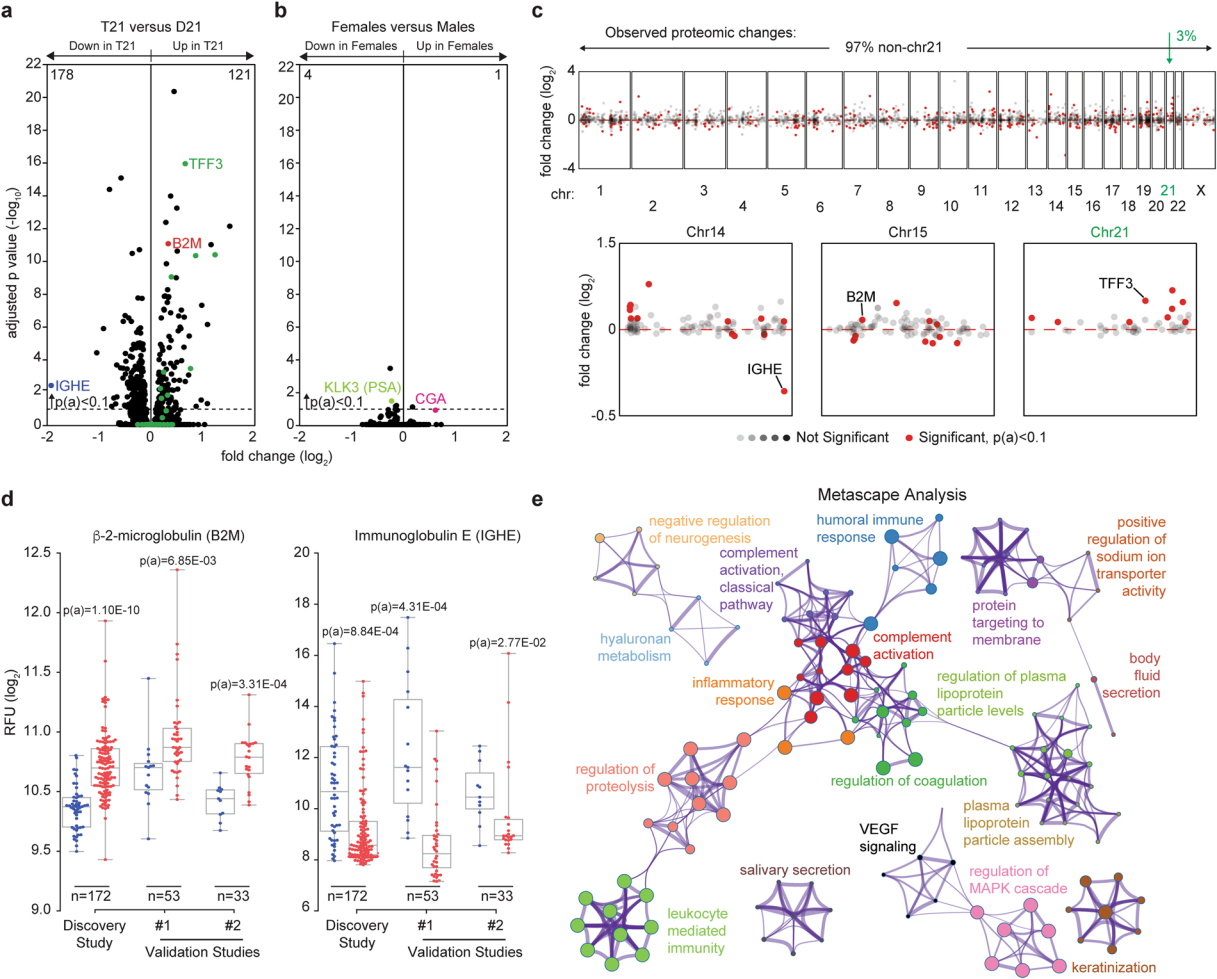
Individuals with trisomy 21 display consistent differences in their circulating proteomes. (**a**) Volcano plots displaying the results of a SOMAscan^®^ proteomics analysis for 3,585 epitopes detected in plasma samples from individuals with or without trisomy 21 (T21). This cohort, referred to as the Discovery Study, involved 120 individuals with T21 and 52 euploid (D21) controls. Adjusted p-values [p(a)] were generated with the Kolmogorov-Smirnov test using a Bonferroni correction for multiple hypothesis testing. When using a cut-off of p(a) <0.1, 178 proteins were identified as significantly downregulated in people with T21 (e.g. Immunoglobulin E, IGHE) versus 121 upregulated proteins (e.g. β-2-microglobulin, B2M). Green dots indicate the 50 proteins encoded in chromosome 21 (chr21) for which aptamers were available in the SOMAscan^®^ assay, only 9 of which passed the p(a) <0.1 cut-off (e.g. TFF3). **(b)** For comparison purposes, data in (A) was re-analyzed to identify differences between females and males. KLK3, downregulated in females, is the prostate-specific antigen (PSA). CGA, upregulated in females, is the alpha subunit of the follicle stimulating and luteinizing hormones. **(c)** Manhattan plot of all detected proteins showing that most differential proteins observed are not encoded on chr21 (top). Individual Manhattan plots showing the proteins encoded on chr14, chr15 and chr21 are shown at the bottom. Red dashed line indicates a zero-fold change. Significantly different proteins are defined as p(a) < 0.1 using KS test with Bonferroni correction. **(d)** Box and whisker plots showing the comparative results for B2M and IGHE in the Discovery Study and two smaller Validation Studies. Adjusted p-values shown from KS test with Bonferroni correction for the Discovery Study and Benjamini-Hochberg for the smaller Validation Studies. See also Figs 1S1 and S2. (**e**) Metascape analysis of significantly differential proteins in the Discovery Study, as defined by p(a) <0.1, 299 proteins in total. Each node represents a GO term, KEGG pathway, or Reactome gene set. The 3,585 proteins detected by SOMAscan^®^ assay were used as the background gene set. See also Figs 1S3, 1S4, and Supplementary File 2.

In order to explore differences in the circulating proteomes of individuals with T21 relative to euploid (D21) individuals, we completed a series of studies using SOMAmer^®^ (*Slow Off-rate Modified Aptamer*) technology, which focuses largely on secreted proteins and those with extracellular domains^18^. We performed a Discovery Study that employed SOMAmer^®^ reagents targeting 3,585 epitopes using plasma from 172 individuals (120 T21 and 52 D21). This cohort was sex-balanced and ranged in age from six months to 21 years (Supplementary File 1). Overall, 299 proteins were differentially detected between the plasma of individuals with T21 versus euploid controls, as defined by the Kolmogorov-Smirnov (KS) test with Bonferroni-adjusted p-values [p(a)] < 0.1 (Fig. 1a, 178 proteins downregulated in individuals with T21, 121 upregulated, Supplementary File 1). For reference, we re-analyzed the data through a comparison of all female versus all male participants in the study, regardless of T21 status. This exercise revealed that changes associated with T21 are far more profound, both in number of proteins affected and fold changes, than those associated with the sex-specific karyotypes (Fig. 1b). Although sex-specific proteins such as KLK3 (kallikrein-related peptidase 3, also known as prostate specific antigen, PSA) and CGA (glycoprotein hormones, alpha polypeptide, the alpha subunit of the follicle stimulating hormone and the luteinizing hormone) partitioned as expected, only five proteins in total were differentially detected in females versus males. To define how many of the protein changes observed in people with DS could be explained by mere increase in gene dosage due to the trisomy, we identified those proteins encoded on chr21. Among the 50 proteins encoded on chr21 for which aptamers were available, 9 were significantly upregulated, and none significantly downregulated (green dots in Fig. 1a, e.g. TFF3), consistent with other genomics studies showing that only a fraction of chr21 genes are significantly upregulated in any particular system^17,19,20^. Thus, most of the proteome changes observed in people with DS correspond to proteins encoded elsewhere in the genome, revealing the existence of signaling pathways consistently dysregulated downstream of T21 (see Manhattan plot in Fig. 1c, top). Examination of individual chromosomes did not reveal any obvious spatial relationships among deregulated proteins, with the notable exception of proteins encoded on chr21, which display a consistent trend toward upregulation (Fig. 1c, bottom).

To assess the degree of reproducibility of these findings, we completed two smaller Validation Studies, measuring 1,047 epitopes in plasma from 53 individuals (Validation Study #1, 38 T21 and 15 D21) and serum from 33 individuals (Validation Study #2, 22 T21 and 11 D21), respectively (Fig. 1 S1a, Supplementary File 1). The use of both plasma and serum samples strengthens our validation efforts by ensuring that changes detected are independent of the presence of clotting factors. We examined the 1047 proteins that were commonly measured in the three studies, and compared those that were significantly different in the Discovery Study, as defined by the more stringent cut-off of a Bonferroni-adjusted p(a) < 0.1, to those with a false discovery rate (FDR) <10% (KS test with Benjamini-Hochberg corrected p-value < 0.1, to account for the smaller sample size) in the two Validation Studies. Using this criterion, we found that ~72% of the differentially abundant proteins from the Discovery Study that were measured in all three studies validated in at least one Validation Study (Fig. 1 S1, Supplementary File 1). Examples are provided in Fig. 1d for β-2-microglobulin (B2M, a subunit of the Major Histocompatibility Complex I (MHC I)), which is consistently elevated in the circulating proteome of people with T21, and Immunoglobulin E (IGHE), which is consistently downregulated (see also Fig. S1). As discussed in more detail below, the changes in both B2M and IGHE can be explained by increased interferon signaling^21–24^. Importantly, replicate variability was minimal both for samples run in replicate within the same assay or a year apart (Fig. 1 S2). Select proteins of interest, such as B2M and CST3 (cystatin C) were confirmed by Western blot using samples from the low and high SOMAmer^®^ detection range (Fig. 1 S2). The lower levels of IgE in people with DS were confirmed by ELISA (Fig. 1 S2).

Having identified consistent and reproducible changes in the circulating proteome of people with DS, we next interrogated functional associations among the 299 differential proteins in the Discovery Study using Metascape software, with the 3,585 proteins measured by SOMAscan^®^ as the background gene set. Metascape queries numerous databases (e.g., GO Biological Processes, KEGG Pathways, Reactome Gene Sets) to find enriched processes in gene lists as well as associations among enriched processes^25^. These Metascape results were dominated by functional categories related to control of the immune system, including humoral immune response, inflammatory response, complement activation, regulation of coagulation, regulation of proteolysis, and leukocyte mediated immunity, among others. (Figs 1e, 1 S3, and Supplementary File 2). The specificity of this result was confirmed by randomly selecting 10 sets of 300 genes from the list of 3,585 SOMAmer^®^ reagents and repeating the Metascape analysis, which revealed that the functional categories identified are not due to an underlying bias in the list of SOMAmer^®^ reagents (Supplementary File 2). Further examination revealed four broad categories encompassing 215 proteins, with some overlapping proteins among groups: Immune Control, Complement and Coagulation, Growth Factor Signaling, and Regulation of Neurogenesis (Fig. 1 S4). Here, we discuss key findings representative of these major functional groups and their potential relationship to the biological impacts of T21.

### People with Down syndrome display proteome changes indicative of chronic autoinflammation

**Figure 2:**
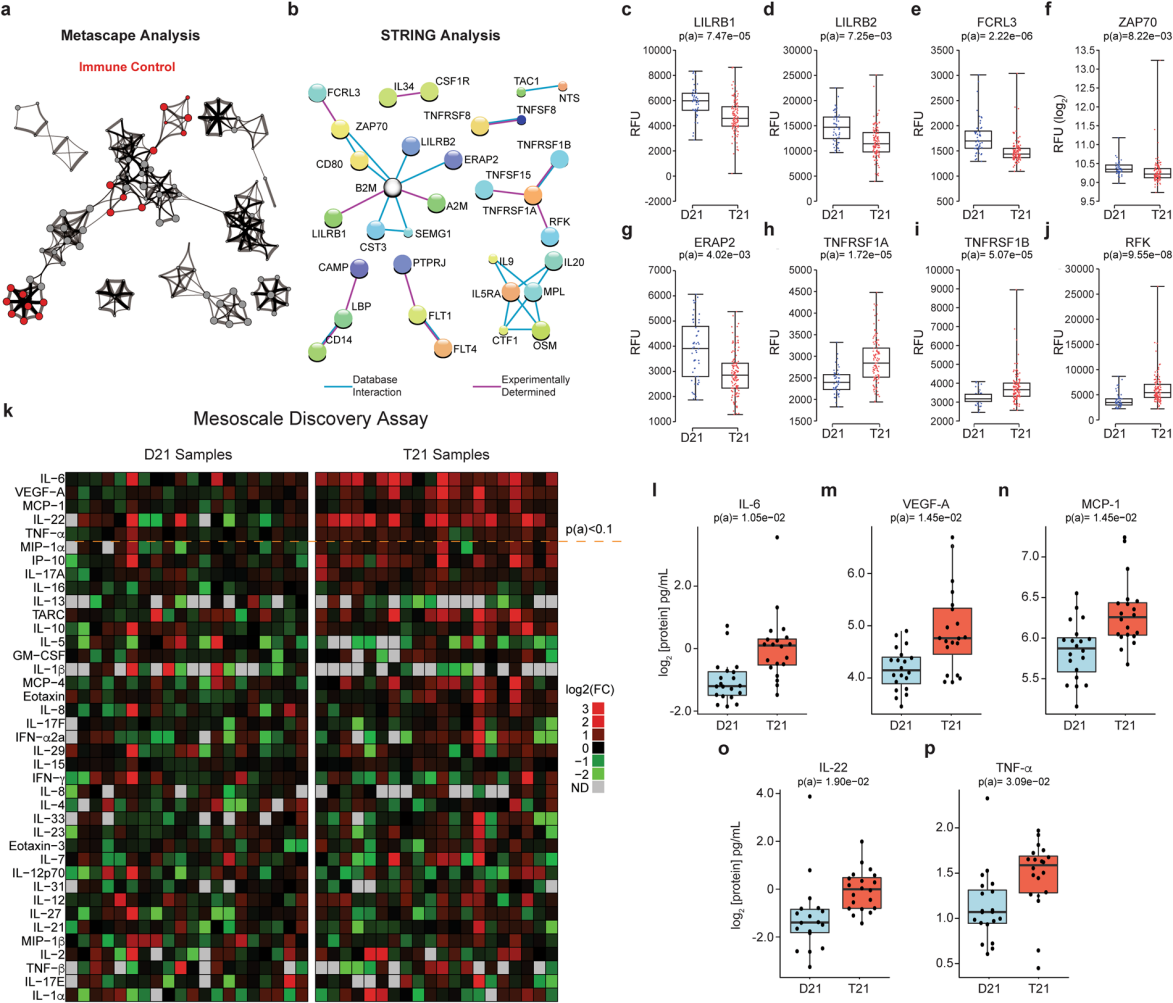
People with Down syndrome show a proteomic signature indicative of profound immune dysregulation. (**a**) Metascape analysis highlighting interconnected protein networks involved in Immune Control among the differential proteins in the Discovery Study. (**b**) STRING analysis identifies interacting groups of deregulated proteins within the Immune Control functional category. **(c–j**) Box and whisker plots of SOMAscan^®^ data for individual proteins deregulated in individuals with T21. p(a) values from KS test with Bonferroni correction. (**k**) Heatmap displaying the results of a Mesoscale Discovery Assay (MSD) measuring the levels of 38 inflammatory markers in a cohort of 40 adult individuals, 20 of them with T21. Proteins are ranked from the most significantly different (top, IL-6) to not significantly different (bottom, IL-1α). The horizontal dashed line marks the p(a) <0.1 cut-off. (**l**–**p**) Box and whisker plots for the top five most different inflammatory markers detected in the MSD assay, all of which are elevated in people with DS. p(a) values from KS test with Benjamini-Hochberg correction.

We first focused on the Immune Control category due to the increased prevalence of autoimmune diseases, myeloproliferative disorders, and leukemias in individuals with T21, and because several independent reports have identified differences in immune signaling via transcriptome analysis of cells and tissues from individuals with T21 and mouse models of DS^1,17,26^. Furthermore, Immune Control constituted the largest functional category, consisting of 150 differential proteins (highlighted in red in Figs 2a and 1 S4). We next queried the STRING database of protein-protein interactions^27^, which revealed numerous groups of physically interacting proteins within this functional category (Fig. 2b). The largest group of physically interacting proteins included factors with key roles in antigen presentation, autoimmunity, and T cell regulation, including B2M, LILRB1, LILRB2, FCRL3, ZAP70, and ERAP2, many of which have been linked to autoimmune disorders (Fig. 2b–g and Table 1).

**Table 1:** Medical conditions associated with differentially abundant proteins identified in this study.

Protein	Condition	Reference
FCRL3	autoimmune thyroid disease	30
FCRL3	rheumatoid arthritis	30
FCRL3	systematic lupus erythematosus	30
FCRL3	neuromyelitis optica	32
Complement (C1QA, C1S, C1R, C3, C6)	Alzheimer’s disease	73–75
Complement (C1QA, C1S, C1R, C3, C6)	systematic lupus erythematosus	63,65
Complement (C1QA, C1S, C1R, C3, C6)	type I interferonopathies	64
IL-10	systematic lupus erythematosus	50
IL-17	systematic lupus erythematosus	52
IL-6	Sjogren’s syndrome	56
IL-6	rheumatoid arthritis	57
IP-10	Aicardi-Goutieres Syndrome	59
M1 Amiopeptidases (ERAP2)	ankylosing spondylitis	44
M1 Amiopeptidases (ERAP2)	inflammatory bowel disease	44
M1 Amiopeptidases (ERAP2)	Behçet’s disease	44
M1 Amiopeptidases (ERAP2)	autoimmune type 1 diabetes	48
M1 Amiopeptidases (ERAP2)	psoriasis	44,45
TNF-α	systematic lupus erythematosus	50
TNF-α	Down syndrome	53
TNF-α	Sjogren’s syndrome	56
TNF-α	rheumatoid arthritis	57
TNF-α	Aicardi-Goutieres Syndrome	58
TNFRSF1A	systematic lupus erythematosus	51
TNFRSF1B	systematic lupus erythematosus	51
VEGF	Aicardi-Goutieres Syndrome	60
ZAP70	severe combined immunodeficiency	38
ZAP70	immunodeficiency 48	39
ZAP70	Infantile-Onset Multisystem Autoimmune Disease 2	40
ZAP70	autoimmune arthritis (mice)	41
FGFR1	myeloproliferative disorders	78
FGFR1	craniofacial abnormalities	76,77
RET	Hirschsprung’s disease	84

B2M is a subunit of the MHC class I complex required for peptide loading and is upregulated in individuals with T21 (Fig. 1d)^28,29^. *B2M* is a known interferon stimulated gene (ISG)^21–23^, and the plasma levels of B2M have been shown to increase during therapeutic administration of IFN-α^23^. LILRB1 and LILRB2 (leukocyte immunoglobulin-like receptors B1 and 2), which are downregulated in individuals with T21 (Fig. 2c,d), are negative regulators of MHC class I signaling^30^. Therefore, increased B2M and decreased LILRBs could be indicative of a chronically active immune system in DS, leading to enhanced antigen presentation, increased signaling downstream of MHC class I engagement, and potential predisposition to autoimmunity.

FCRL3, which is significantly downregulated in people with DS (Fig. 2e), is a member of the immunoglobulin receptor superfamily and one of several Fc receptor-like glycoproteins involved in immune control^31^. Importantly, mutations and polymorphisms in this gene have been associated with autoimmune thyroid disease, rheumatoid arthritis, and systemic lupus erythematosus (SLE)^32–34^. ZAP70 (Src-related kinase, SRK), which is also downregulated in people with T21 (Fig. 2f), is a tyrosine kinase that physically interacts with both FCRL3 and the zeta-chain (CD247) of the T cell receptor (TCR)^35,36^. ZAP70 regulates motility, adhesion, and cytokine expression of mature T cells, as well as thymocyte development^37–39^. Mutations in ZAP70 have been associated with the development of autoimmune and autoinflammatory diseases, including Immunodeficiency-48 (IMD48) and Infantile-Onset Multisystem Autoimmune Disease 2^40–42^. In mice, missense mutations in ZAP70 cause chronic autoimmune arthritis^43^. ERAP2, also downregulated in people with DS (Fig. 2g), is a member of the M1 aminopeptidase family involved in trimming antigenic epitopes for presentation by MHC class I molecules^44,45^. Several genome-wide association studies have linked these aminopeptidases to a range of immune-mediated diseases such as psoriasis, ankylosing spondylitis, inflammatory bowel disease, Behçet’s disease, and type I diabetes^46–50^. Therefore, deregulation of FCRL3, ZAP70 and ERAP2 could potentially contribute to the high predisposition to autoimmune conditions in the population with DS.

Another key result from this analysis is that people with DS show obvious signs of increased TNF-α signaling. The SOMAscan^®^ proteomics platform revealed elevated circulating levels of the two TNF-α receptors, TNFRSF1A and TNFRSF1B, as well as the protein kinase RFK which is required for ROS production downstream of TNF-α receptor engagement (Fig. 2h–j)^51^. Higher levels of circulating TNFRSF1A were confirmed in both validation studies (Fig. 2 S1). As described below, people with DS also display higher levels of the TNF-α ligand.

Given the potential importance of inflammatory cytokines in driving DS-associated phenotypes, we expanded on the SOMAscan^®^ results by measuring a set of 38 cytokines using a different platform, the Mesoscale Discovery assay (MSD), in a separate cohort of 40 adult participants (ages 20–65, Supplementary File 1). These efforts revealed that individuals with DS have significantly increased levels of potent inflammatory cytokines and chemokines known to act downstream of IFN signaling, including IL-6, IL-22, MCP-1 (CCL2), TNF-α, and VEGF-A, (Fig. 2k–p, Supplementary File 1). Another inflammatory cytokine that was also significantly elevated in people with DS in each of the three SOMAscan^®^ studies, but which fell below the statistical significance cut-off in the MSD assay was CCL17 (TARC) (Fig. 2 S1). Notably, many of these factors are upregulated in the circulating proteomes of individuals with chronic inflammatory diseases (Table 1). For example, levels of TNF-α, the two soluble TNF-α receptors, as well as IL-10 and IL-17, are all increased in individuals with SLE, a condition associated with hyperactive IFN signaling^52–54^, and at least one previous report found increased TNF-α levels in the serum of individuals with T21^55^. IL22, which is an IL-10 family cytokine related to IFN-γ, is elevated in the serum of patients with psoriasis and known to contribute to disease etiology^56,57^. TNF-α and IL-6 are clearly elevated in people with rheumatoid arthritis and Sjogren’s syndrome^58,59^. Additionally, TNF-α, IP-10, and VEGF, were found to be upregulated in the cerebrospinal fluid or plasma from individuals with Aicardi-Goutieres Syndrome (AGS), a canonical monogenic Type I Interferonopathy^60^.

In order to assess how many of the protein changes could be linked to interferon signaling, we mined the Interferome database (www.interferome.org), and found a statistically significant enrichment of interferon-related proteins among those that are differentially detected in people with T21 relative to all proteins detected by the SOMAscan^®^ platform (p = 1.91E-03, hypergeometric test, bolded in Fig. 1 S4). Many other observed changes could be indirectly linked to increased interferon signaling. For example, the strikingly decreased levels of IgE (Figs 1d, 1 S2) could be explained by the established role for IFN-α/β in suppressing allergic inflammatory processes by preventing granulocyte activation and IL-4-mediated isotype switching to IgE^24^. Another example is cystatin 3 (CST3), which is clearly elevated in people with DS (Fig. 1 S2). Elevated circulating levels of CST3 are indicative of decreased glomerular filtration^61^, and hyperactive interferon signaling is known to affect renal function during lupus nephritis^62^, and during viral glomerulonephritis^63^.

In sum, these results reveal a clear pro-inflammatory signature in the circulating proteome of people with DS, with multiple ties to interferon signaling.

### Trisomy 21 causes hypocomplementia

**Figure 3:**
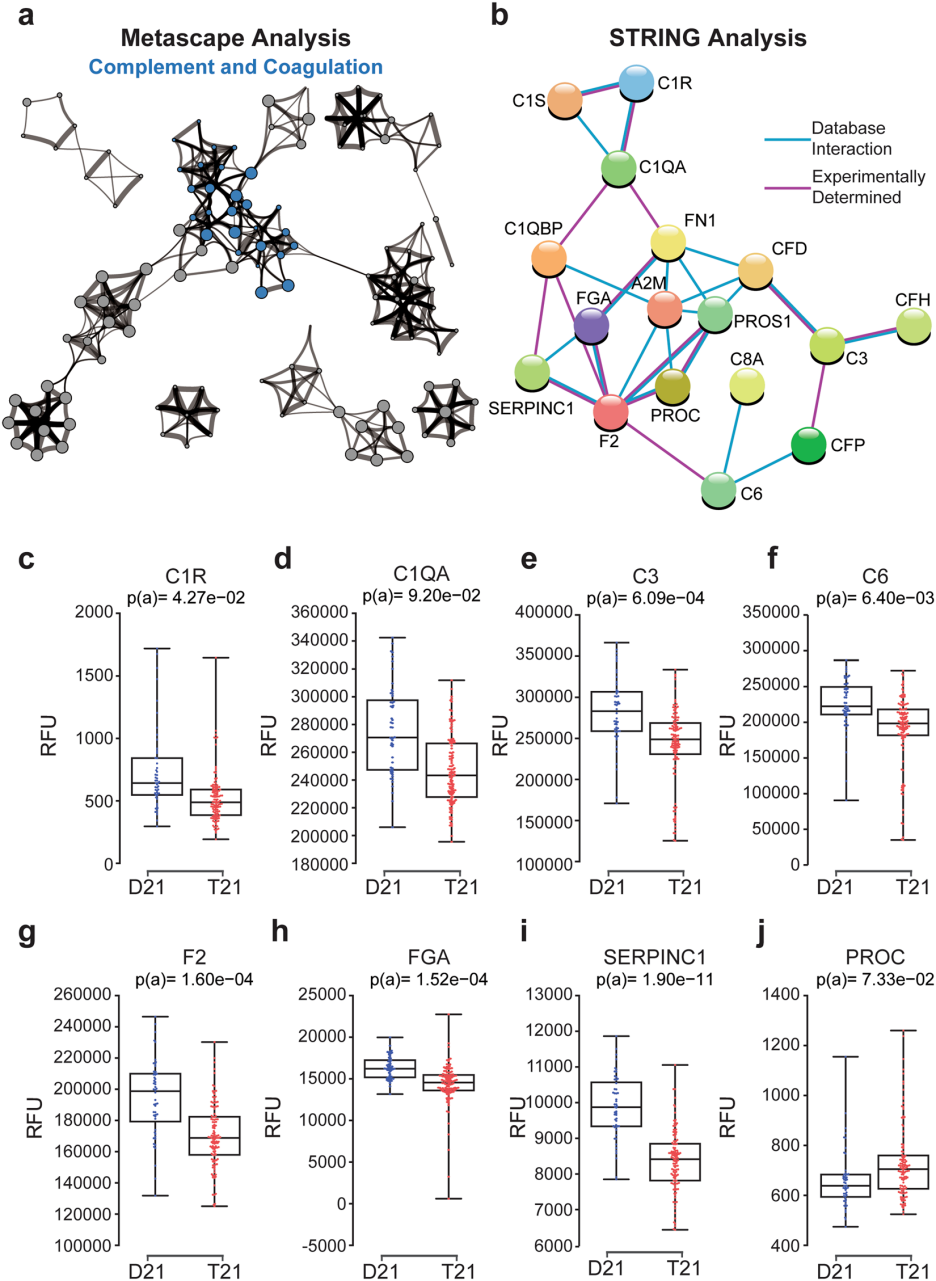
People with Down syndrome exhibit hypocomplementia. (**a**) Metascape analysis highlighting interconnected protein networks related to Complement and Coagulation among the differential proteins in the Discovery Study. (**b**) STRING analysis identifying interacting groups of deregulated proteins within the Complement and Coagulation functional category. (**c–j**) Box and whisker plot showing data for individual proteins. p(a) values from KS test with Bonferroni correction.

Another category of proteins identified by Metascape and STRING analyses as deregulated in individuals with T21 is the Complement and Coagulation cascade (Figs 1e and 3a,b). This group consists of 21 proteins, 18 of which are downregulated in the circulating proteome of people with DS, suggesting a complement consumption phenotype. Examples of subunits of the complement pathway that are downregulated in individuals with T21 include C1R, C1QA, C3, and C6 (Fig. 3c–f). The reproducibility of this result is further illustrated by the consistent depletion of C1S observed in all three studies (Fig. 3 S1).

Hypocomplementia is a hallmark of immune complex diseases, a diverse group of inflammatory conditions characterized by antigen–antibody deposition and consequent complement activation^64^, including interferon-driven conditions such as SLE^65^. In fact, hypocomplementia is a common feature of type I interferonopathies^66,67^, suggesting that chronic activation of interferon signaling could cause complement consumption. Hypocomplementia could clearly contribute to the observed increased risk of bacterial lung pneumonia and *otitis media* in the population with DS^68^, as complement depletion prevents proper clearance of *Streptococcus pneumoniae* ^69^. The kidney is particularly susceptible to complement-mediated injury^70^, and CST3, B2M, and TFF3, which are among the most significantly upregulated proteins in DS (Supplementary File 1), have all been used to monitor kidney function, as their plasma levels increase upon kidney injury^71^. As life expectancy increases for individuals with DS, kidney problems are becoming more apparent^72^, and we hypothesize that this could be due to accumulation of immune complexes and complement activation in the kidney. There are also clear links between the complement system and Alzheimer’s disease (AD). People with DS are the largest human population with a genetic predisposition to early-onset AD, which is largely explained by the presence of the *APP* gene on chr21^73^. However, several complement subunits also play key roles in microglia-mediated synapse destruction during AD progression^74–76^, and both beta amyloid deposits and soluble B2M are known to activate the classic complement cascade^77^. Thus, we posit that hypocomplementia could modulate AD progression in these individuals. Finally, multiple proteins within the coagulation cascade were also identified as downregulated in T21 samples, including Factor II (F2), fibrinogen (FGA) and the inhibitor SERPINC1 (Fig. 3g–i), while another inhibitor of numerous coagulation factors, protein C (PROC), is upregulated (Fig. 3j).

### Trisomy 21 causes changes in circulating levels of growth factor receptors and proteins involved in neurogenesis

**Figure 4:**
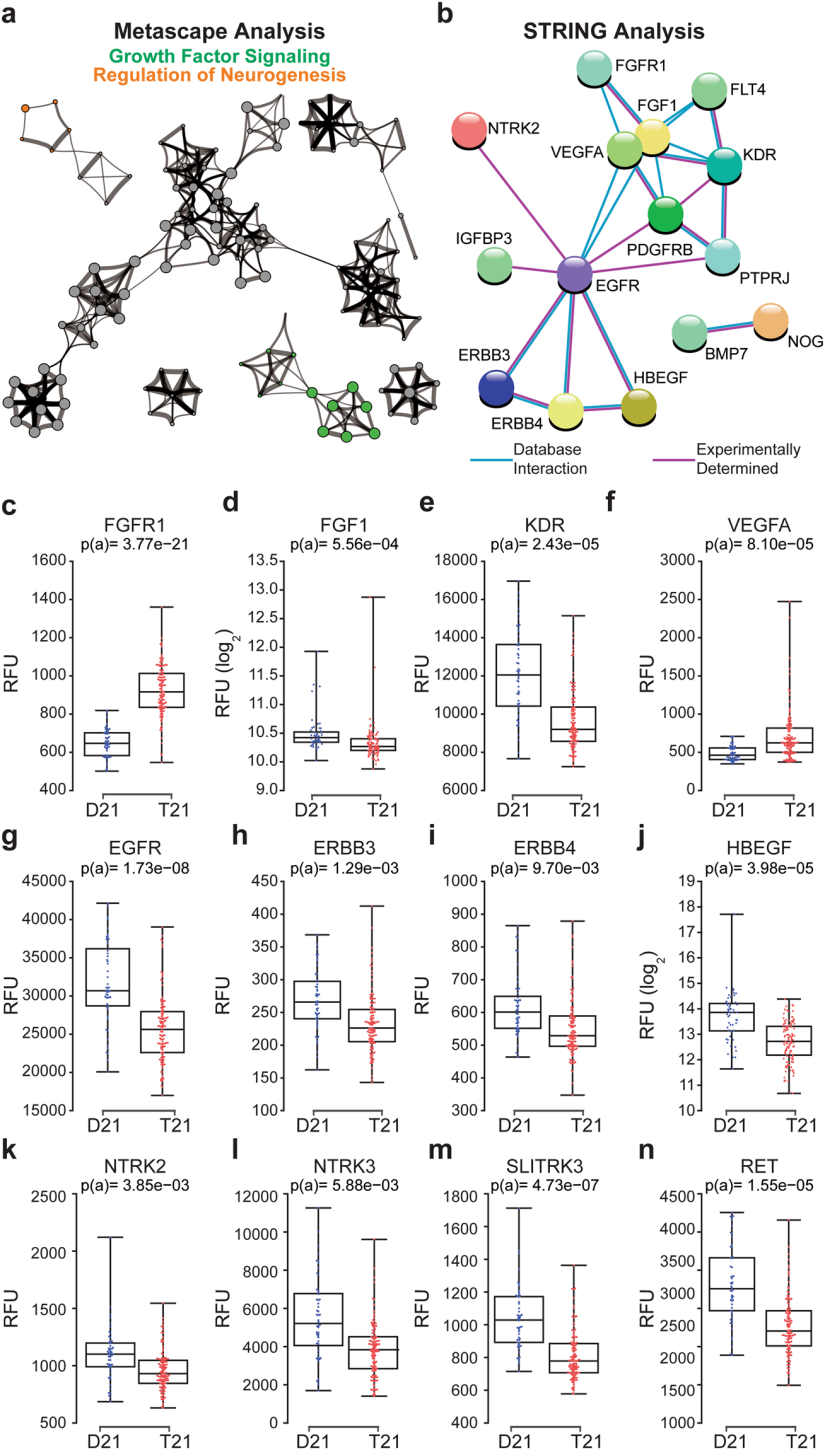
People with Down syndrome show different levels of proteins involved in growth factor signaling and control of neurogenesis. (**a**) Metascape analysis highlighting protein networks related to Growth Factor Signaling and Regulation of Neurogenesis among the differential proteins in the Discovery Study. (**b**) STRING analysis identifying interacting groups of deregulated proteins within the Growth Factor Signaling category. (**c**–**n**) Box and whisker plots displaying SOMAscan^®^ data for individual proteins deregulated in individuals with T21. p(a) from KS test with Bonferroni correction.

Another prominent functional category that Metascape identified as highly deregulated in people with T21 is Growth Factor Signaling (Figs 1e and 4a). Significantly deregulated within the group of Growth Factor Signaling proteins are components of the FGF, VEGF, and EGF pathways (Fig. 4b). For example, FGFR1 is upregulated in individuals with T21, while its ligand FGF1, is down (Fig. 4c,d). Gain-of-function mutations in FGFR1 are associated with myeloproliferative disorders leading to leukemia, as well as hereditary conditions leading to skeletal and craniofacial abnormalities, both of which are common in DS^78–80^. VEGF signaling shows the opposite pattern, with downregulation of the receptor (KDR, VEGFR) and upregulation of the ligand VEGFA (Fig. 4e,f). Of note, VEGFA induction is a hallmark of inflammatory processes leading to wound healing^81^. Interestingly, numerous components of the EGF signaling cascade are downregulated in DS, including the receptors EGFR, ERBB3, and ERBB4, and the ligand itself (HBEGF) (Fig. 4g–j).

Notably, 17 of the 75 proteins linked to Growth Factor Signaling by Metascape were also linked to Regulation of Neurogenesis, including EGFR, ERBB4, NTRK2, NTRK3, and RET (Fig. 1 S4). For example, NTRK2 and NTRK3, the transmembrane receptors for the Brain Derived Neurotrophic Factor (BDNF) and Neurotrophin 3, respectively^82^, are clearly downregulated in the plasma of people with DS (Fig. 4k,l). A third neurotrophin receptor required for normal GABAergic synapse development, SLITRK3^83^, is also downregulated (Fig. 4m). Of note, altered GABAergic signaling has been linked to the cognitive deficits caused by T21 in myriad studies (reviewed in^84^). Finally, RET, a receptor tyrosine kinase of the cadherin superfamily with key roles in neural crest development^85^, also displays lower circulating levels in people with DS (Fig. 4n). Interestingly, loss-of-function mutations in RET cause Hirschsprung’s disease^86^, which is significantly more prevalent in people with DS^87^. Clearly, our proteomics datasets provide many hypothesis-generating results that could enable future mechanistic investigations to decipher the molecular underpinnings of the developmental problems and cognitive deficits observed in DS.

### Final Remarks

Our study of the circulating proteome in people with DS, the largest and most comprehensive study of this kind reported to date, reveals that T21 causes profound immune dysregulation, with many of the changes being highly reminiscent of those observed in type I interferonopathies and other autoinflammatory conditions^54,58–60,88^ (Table 1). These results provide a new framework for understanding the etiology of DS-associated co-morbidities and the developmental phenotypes of DS. Most importantly, these findings reinforce the notion that individuals with DS could benefit from therapeutic strategies that diminish interferon signaling.

## Materials and Methods

### Study population and sample collection

The study was approved by the Colorado Multiple Institutional Review Board (COMIRB #11-1790 for the three cohorts analyzed with the SOMAscan^®^ proteomics platform, and COMIRB #15-2170 for those samples analyzed with MSD). Written informed consent was obtained from parents or guardians of each participant, and assent was obtained from participants over the age of 7 who were cognitively able to assent. All procedures were performed in accordance with COMIRB guidelines and regulations. Cohort information can be found in Supplementary File 1. Discovery Study - Plasma samples from children with DS (120) and healthy controls (52), in some cases siblings of T21-affected individuals, were provided by the Sie Center for Down Syndrome, a coordinated care clinic at the Children’s Hospital Colorado that focuses specifically on clinical care for children with DS. Additional control samples were obtained from the Diabetes Auto Immunity Study in the Young (DAISY) study^89^. Validation Study #1 - Additional plasma samples from individuals with DS were obtained from the Sie Center and additional healthy controls obtained from the Translational Nexus (COMIRB #08-1276). Validation Study #2 - Additional serum samples from children with DS and healthy controls were provided by the Translational Nexus. MSD Study - Plasma samples were obtained from The Human Trisome Project Biobank (COMIRB #15-2170, www.trisome.org). Serum and plasma were collected in Vacutainer tubes (BD, SST - gold capped, and K2, EDTA - purple capped). Serum was allowed to clot for 30 minutes at room temperature and then spun at 1,200 × g for 10 minutes at 4 °C. Serum was aliquoted and stored at −70 °C. Plasma was treated the same as serum except for the clotting step, which was intentionally omitted.

### SOMAscan^®^ assay

The SOMAscan^®^ assay has been described in detail previously^90–93^. Briefly, each of the 3,585 SOMAmer^®^ reagents (Discovery Study) and 1,047 SOMAmer^®^ reagents (Validation Studies) binds a target protein and is quantified on a custom Agilent hybridization chip. Normalization and calibration were performed according to SOMAscan^®^ Data Standardization and File Specification Technical Note (SSM-020). The output of the SOMAscan^®^ assay is reported in relative fluorescent units (RFU). These data were log-transformed and the non-parametric Kolmogorov-Smirnov (KS) test was used to identify proteins that were differentially detected in samples from people with and without DS. Multiple hypothesis correction was performed using the Bonferroni method for the larger discovery study or the Benjamini-Hochberg method for the smaller Validation Studies.

### MSD assay

Mesoscale Discovery V-PLEX 29-PLEX Human Cytokine and a U-PLEX custom array were combined to detect 38 cytokines in plasma from 40 individuals (20 D21 and 20 T21). Assays were performed according to manufacturer’s instructions. These data were log-transformed and the non-parametric Kolmogorov-Smirnov (KS) test was used to identify proteins that were differentially expressed in the DS and control study populations. Multiple hypothesis correction was performed using the Benjamini-Hochberg method.

### IgE ELISA

Plasma IgE levels were tested using the Human IgE SimpleStep ELISA Kit (Abcam ab195216) per manufacturer’s instructions. Briefly, 50 μL of a 1:400 dilution of plasma EDTA was incubated with antibody cocktail (1X capture antibody and 1X detector antibody-HRP) for one hour at room temperature, washed three times and incubated with substrate for 10 min at room temperature. Absorbance was then determined at 450 nm. Each sample was assayed in duplicate and absolute values determined using linear regression analysis with standard curves.

### Data Availability

All data generated or analyzed during this study are included in this published article (and its Supplementary Information files).

## Electronic supplementary material

Supplementary Information

Supplementary File 1

Supplementary File 2
