# Supplementary material for: Trisomy 21 causes changes in the circulating proteome indicative of chronic autoinflammation
Source: Sci Rep. 2017 Nov 1;7:14818. doi: 10.1038/s41598-017-13858-3 (PMC5665944; doi:10.1038/s41598-017-13858-3)
Supplement: Supplementary file 1 — Supplementary Information [file 41598_2017_13858_MOESM1_ESM.pdf]

Trisomy 21 causes changes in the circulating proteome indicative of chronic autoinflammation

Kelly D. Sullivan[1,2], Donald Evans[1], Ahwan Pandey[1,2], Thomas H. Hraha[3], Keith P. Smith[1], Neil Markham[1], Angela L. Rachubinski[4], Kristine Wolter-Warmerdam[5], Francis Hickey[5], Joaquin M. Espinosa[1,2,6*] and Thomas Blumenthal[1,6,7*]

[1] Linda Crnic Institute for Down Syndrome, University of Colorado School of Medicine, Aurora, Colorado 80045, USA

[2] Department of Pharmacology, University of Colorado School of Medicine, Aurora, Colorado 80045, USA

[3] SomaLogic, Inc., Boulder, CO 80301, USA

[4] JFK Partners/Developmental Pediatrics, Department of Pediatrics, University of Colorado School of Medicine, Aurora, Colorado 80045, USA

[5] Anna and John J. Sie Center for Down Syndrome, Children's Hospital Colorado, Aurora, Colorado, 80045, USA

[6] Department of Molecular, Cellular and Developmental Biology, University of Colorado Boulder, Boulder, Colorado 80203, USA

[7] Department of Biochemistry and Molecular Genetics, University of Colorado School of Medicine, Aurora, Colorado 80045, USA

*Correspondence to: joaquin.espinosa@ucdenver.edu / thomas.blumenthal@ucdenver.edu

Supplementary Files.

Supplementary File 1. (a) Discovery Study. Kolmogorov Smirnoff analysis of T21 versus D21 cohort. Columns include: (A) Gene ID, (B) Chromosome, (C) Chromosome Start Position, (D) Chromosome End Position, (E) Chromosome Strand, (F) Gene Length, (G) Uniprot ID, (H) Soma ID, (I) Seq ID, (J) Average RFU, (K) Euploid (D21) average RFU, (L) Trisomy 21 (T21) average RFU, (M) Fold change (T21/D21), (N) Log_2 fold change, (O) p-value, (P) Bonferroni (Bon) adjusted p-value. **(b) Validation Study #1.** Kolmogorov Smirnoff analysis of T21 versus D21 cohort. Columns include: (A) Gene ID, (B) Uniprot ID, (C) Soma ID, (D) Seq ID, (E) Average RFU, (F) D21 average RFU, (G) T21 average RFU, (H) Fold change (T21/D21), (I) Log_2 fold change, (J) p-value, (K) Benjamini-Hochberg (BH) adjusted p-value. **(c) Validation Study #2.** Kolmogorov Smirnoff analysis of T21 versus D21 cohort. Columns include: (A) Gene ID, (B) Uniprot ID, (C) Soma ID, (D) Seq ID, (E) Average RFU, (F) D21 average RFU, (G) T21 average RFU, (H) Fold change (T21/D21), (I) Log_2 fold change, (J) p-value, (K) Benjamini-Hochberg (BH) adjusted p-value. **(d) Study overlaps.** Lists of differential proteins common among the three SOMAscan® studies. **(e) Meso Scale Discovery Assay.** Kolmogorov Smirnoff analysis of T21 versus D21 cohort. Columns include: (A) Cytokine, (B) D21 median protein concentration (ng/mL), (C) T21 median protein concentration (ng/mL), (D) Fold change (T21/D21), (E) Log_2 fold change, (F) p-value, (G) Benjamini-Hochberg adjusted p-value. **(f) Cohort information.** Statistics on individual cohorts including sex and age.

Supplementary File 2. Metascape Analysis. (a) Annotation of gene list used for analysis. **(b)** Enrichment analysis. **(c)** Random permutation analysis with 10 list of 300 randomly selected proteins.

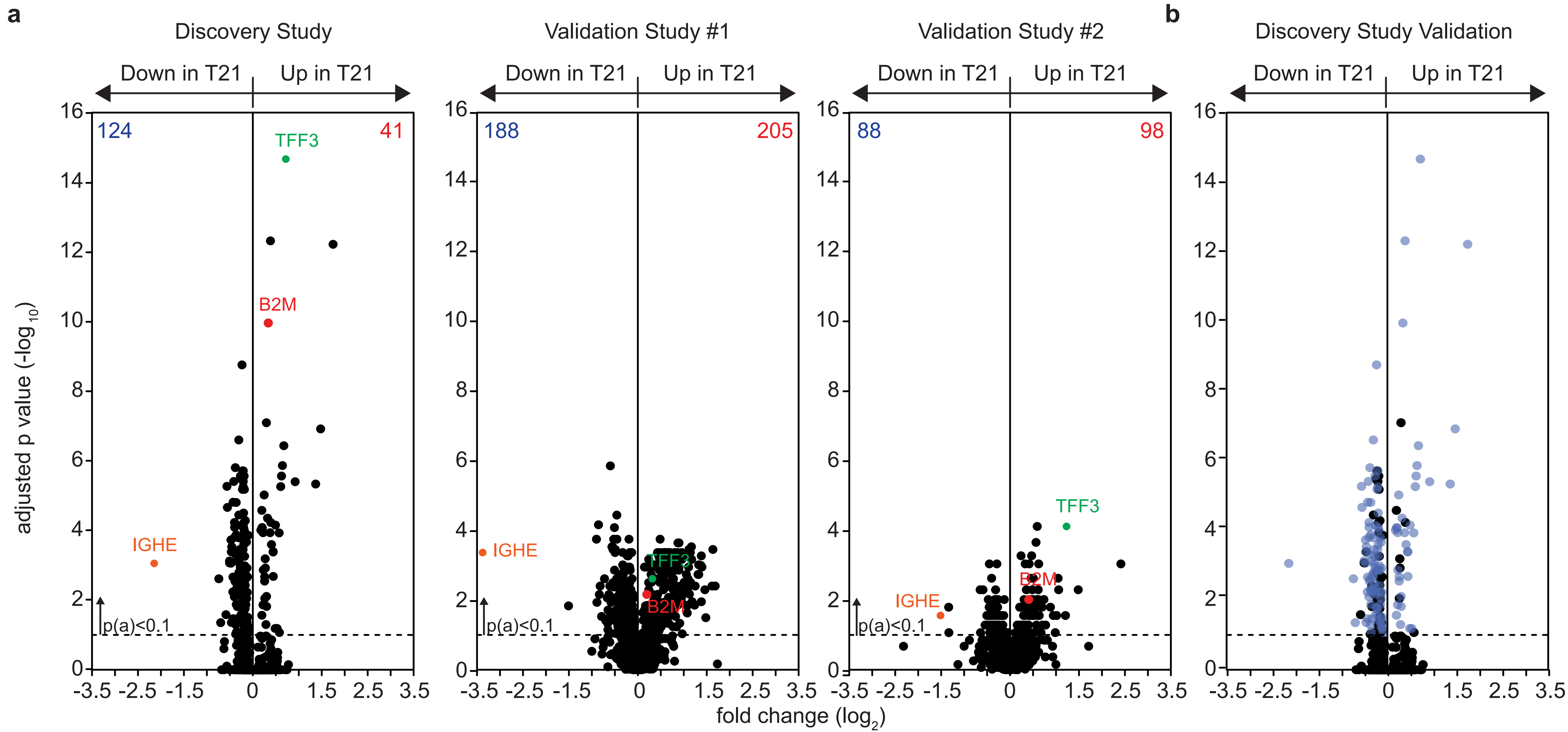

Figure 1 S1. (a) Volcano plots showing the 1047 proteins commonly detected in the samples unique to the Discovery Study (83 T21 and 52 D21), Validation Study #1 (38 T21 and 15 D21), and Validation Study #2 (22 T21 and 11 D21). Adjusted p values were calculated using KS test with Bonferroni correction for the Discovery Study and Benjamini-Hochberg correction for the Validation Studies. **(b)** Volcano plot of the Discovery study highlighting in blue those proteins with p(a)<0.1 (KS test with Bonferroni correction), that validated in one or both Validation Studies p(a)<0.1 (KS test with Benjamini-Hochberg correction).

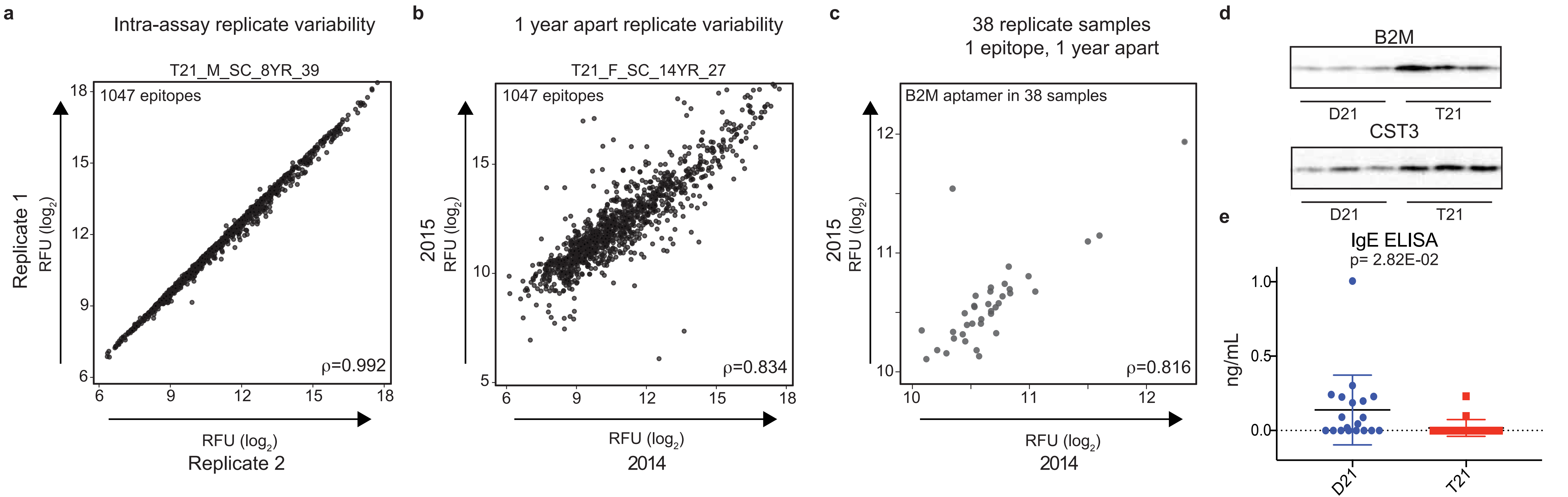

Figure 1 S2. (a). Scatter plot showing the 1047 epitopes measured twice during the same assay for sample T21_M_SC_8YR_39. **(b).** Scatter plot showing 1047 epitopes measured twice, in two different assays a year apart, for sample T21_F_SCP_14YR_27. **(c).** Scatter plot showing the values for the aptamer recognizing B2M in 38 samples that were measured twice, in two different assays a year apart. **(d)** Western blots for B2M and CST3 on select plasma samples from euploid (D21) and T21 individuals that showed low and high signals in the SOMAscan assay. **(e)** ELISA of IgE levels from 19 individuals with T21 and 19 euploid controls. Difference between the groups was calculated using the Kolmogorov-Smirnoff test.

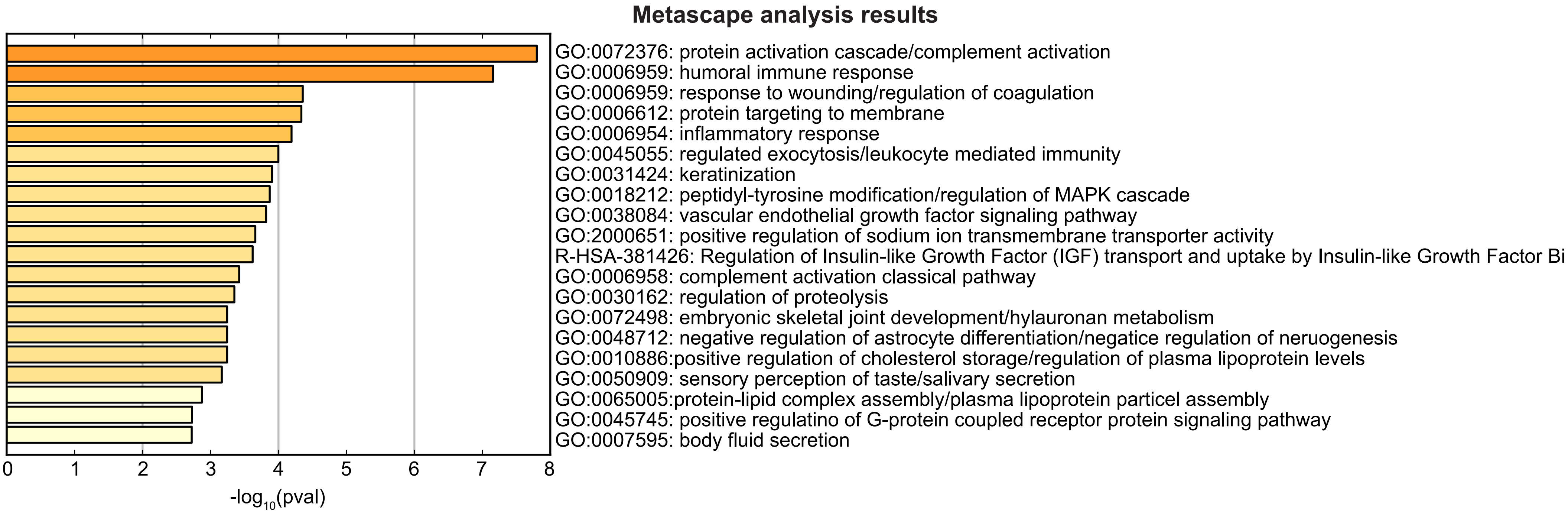

Figure 1 S3. Metascape heatmap of terms enriched in the 299 most significantly different proteins from the Discovery Study, colored by p-values.

Immune Control

**A2M**	**CHP1**	IL34	RABEPK
ADAMTS13	CLEC4M	**IL37**	REG3A
ADGRE5	**COCH**	IL5RA	REN
AHSG	CREG1	IL9	RETN
**ANXA2**	CRLF1,CLCF1	IMPDH1	RFK
**APLP2**	**CSF1R**	**IMPDH2**	**RNASE6**
APOB	**CST2**	ITGA1,ITGB1	**SEMG1**
**APOD**	CST3	ITIH1	**SERPINA3**
**APOE**	**CTF1**	KDR	SERPINA4
APOH	**CTSC**	KIR3DL2	SERPINC1
**B2M**	**CTSH**	**KLK3**	**SIGLEC7**
**BGN**	**CYTL1**	KLK5	**STAB2**
BPIFB1	DEFA1	KLK8	TAC1
**C1QA**	**DLL1**	LBP	**TF**
C1QBP	DSG1	**LILRB1**	**TIMP1**
**C1R**	EFNB1	LILRB2	**TIMP2**
**C1S**	EGFR	LPO	TNFRSF11B
**C3**	**ERAP2**	**LTA4H**	**TNFRSF19**
C6	**F2**	LY6G6C	**TNFRSF1A**
C6orf58	**FAIM3**	MPL	**TNFRSF1B**
C8A	FCN2	**MSR1**	**TNFRSF8**
**CAMP**	FCRL1	MST1R	**TNFSF15**
**CBR3**	FCRL3	MUL1	**TNFSF18**
CCL1	FGA	**NPC2**	TNFSF8
**CCL17**	**FGF1**	**NRP1**	**TYRO3**
CCL28	FKBP1B	**NTRK3**	**VEGFA**
**CCL3L1**	**FLT1**	NTS	**VTN**
**CD14**	FLT4	OAF	**ZAP70**
CD177	**FN1**	**OLR1**	
**CD22**	**GZMB**	**OSM**	
**CD300C**	**HMGB2**	PDGFRB	
**CD33**	**HSPA8**	PGLYRP2	
**CD36**	HTN3	**PI3**	
**CD80**	**HYAL1**	PIGR	
**CD97**	IGHE	PIK3C2A	
**CDK5**	IGHE,IGK,IGL	**PLA2G7**	
**CFD**	IGHM	**PNP**	
**CFH**	IGHM,IG-	PRG3	
CFP	J,IGK,IGL	PROC	
CHGA	IL17F	**PROS1**	
**CHI3L1**	IL20	PTPRJ	

Complement and Coagulation

**A2M**	IGHE
ADAMTS13	IGHM
AHSG	**KLK13**
**ANXA2**	KLK5
**APOD**	KLK6
**APOE**	KLK8
APOH	LBP
**C1QA**	NOG
C1QBP	**NRP1**
**C1R**	**NTRK3**
**C1S**	**OSM**
**C3**	PCSK7
C6	PDGFRB
C8A	PROC
**CBX5**	**PROS1**
CD177	REG3A
**CD36**	REN
CDH3	**SERPINA3**
**CDK5**	SERPINC1
**CFD**	**SFN**
**CFH**	**STAB2**
CFP	TAC1
CLEC4M	**TESC**
CST3	TFF1
**CTSH**	**TFF3**
**CTSV**	TFPI2
**DCBLD2**	**TIMP1**
**DDR1**	**TYRO3**
DSG1	**VEGFA**
EGFR	**VTN**
**ERBB3**	WNK3
ERBB4	C1QA,C1QB,C1QC
**F10**	C8A,C8B,C8G
**F2**	FGA,FGB,FGG
FCN2	**PLA2G7**
FGA	PTGDS
FKBP1B	
**FN1**	
**GZMB**	
**HBEGF**	
**HK2**	

Growth Factor Signaling

**ANXA2**	KDR
**APOE**	MAPKAPK5
AURKB	MST1R
BMP7	MUL1
**C3**	**NELL1**
CCL1	NOG
**CCL17**	NPTN
**CCL3L1**	**NRP1**
CCNH	NTRK2
**CD36**	**NTRK3**
**CD80**	OLFM2
**CDK5**	**OSM**
**CHI3L1**	PAPPA
**CHP1**	PDGFRB
CRLF1	PTPRJ
**CSF1R**	REN
**CTF1**	RET
**DDR1**	**ROR1**
**DLL1**	RPS3
**EFEMP1**	STK16
EFNB1	**TGFBR3**
EGFR	**TIMP2**
**ERBB3**	**TNFRSF19**
ERBB4	**TNFRSF1A**
ERP29	**TNFSF15**
**F10**	**TNFSF18**
**F2**	**TPST2**
FGA	TYRO3
**FGF1**	UBC
**FGFR1**	**VEGFA**
**FLT1**	**VTN**
FLT4	WNK3
**FN1**	**ZAP70**
**HBEGF**	
**IGFBP3**	
IGFBP6	
**IGFLR1**	
IL20	
IL34	
IL5RA	
INHBC	
**ITGA1**	

Regulation of Neurogenesis

AHSG	**TNFRSF8**
**APOE**	VWC2
**B2M**	
BIN1	
BMP7	
CA10	
CBLN4	
**CD80**	
**CDK5**	
CDK5,CDK5R1	
CHGA	
CHGB	
**COCH**	
**DLL1**	
EFNB1	
EGFR	
ERBB4	
EVA1C	
**F2**	
FSTL4	
KLK8	
**NELL1**	
NOG	
NPDC1	
NPS	
NPTN	
**NRP1**	
NTRK2	
**NTRK3**	
NTS	
NXPH1	
**PCDH9**	
**PCDHA4**	
PCDHAC2	
PRG3	
PTGDS	
RET	
**ROR1**	
S100A5	
**SLITRK3**	
TAC1	

Figure 1 S4. Table of differentially abundant proteins in each of the four largest functional categories. Protein identified as interferon-related in the Interferome database are highlighted in bold.

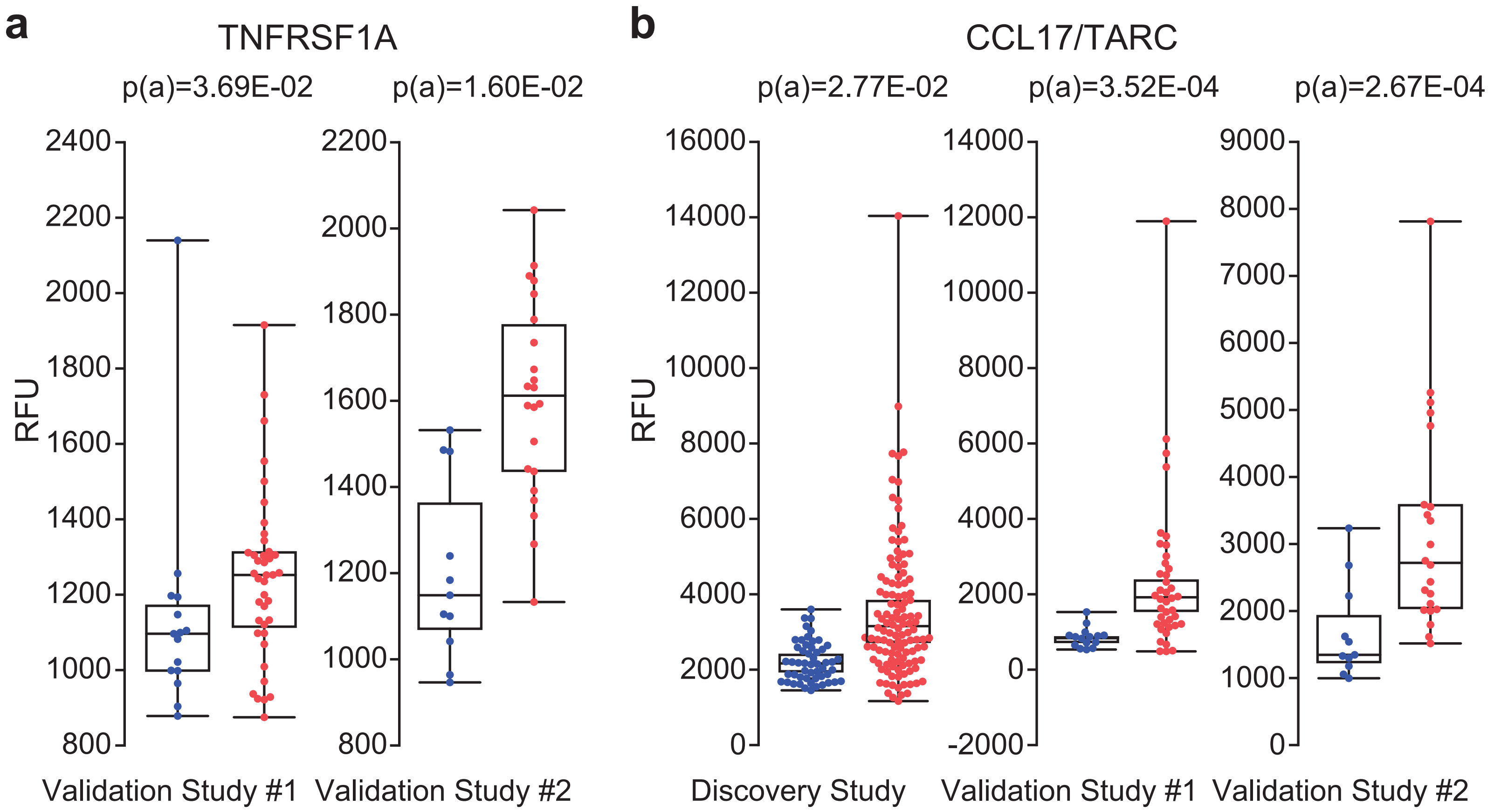

Figure 2 S1. Boxplots of raw data for TNFRSF1A from each validation study (**a**) or all three studies for CCL17/TARC (**b**).

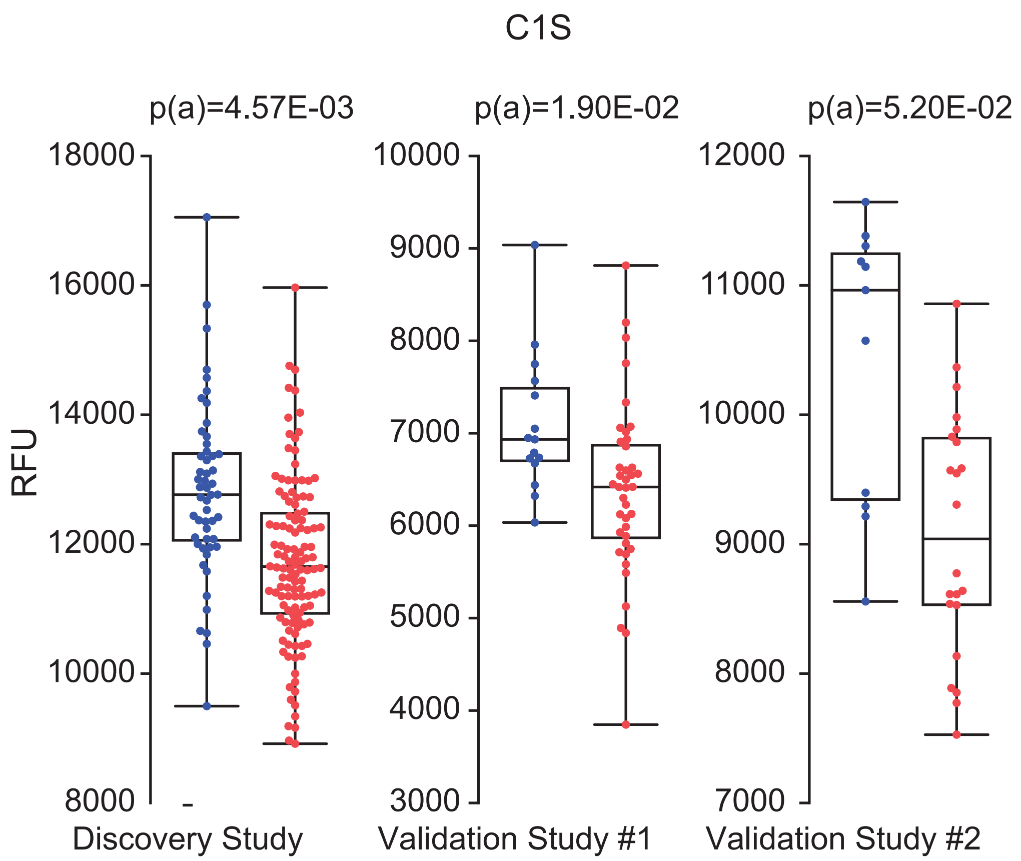

Figure 3 S1. Boxplots of raw data from all three studies for complement subunit C1S.

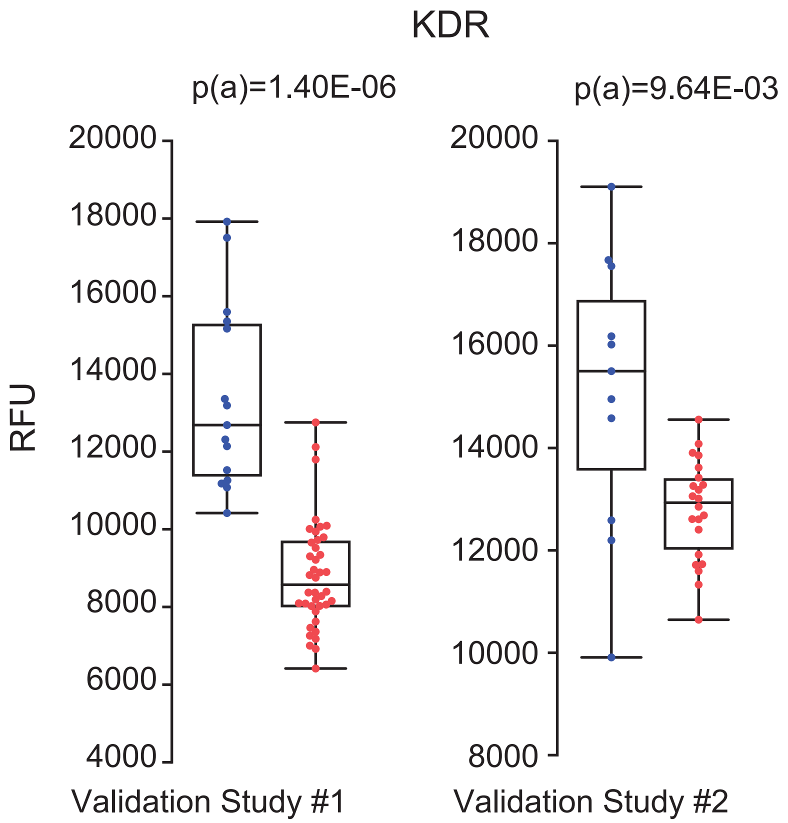

Figure 4 S1. Boxplots of raw data for KDR from each validation study.
